# Albuminuria, Forgotten No More: Underlining the Emerging Role in CardioRenal Crosstalk

**DOI:** 10.3390/jcm13030777

**Published:** 2024-01-29

**Authors:** Gregorio Romero-González, Néstor Rodríguez-Chitiva, Carles Cañameras, Javier Paúl-Martínez, Marina Urrutia-Jou, Maribel Troya, Jordi Soler-Majoral, Fredzzia Graterol Torres, Maya Sánchez-Bayá, Jordi Calabia, Jordi Bover

**Affiliations:** 1 Nephrology Department, Germans Trias i Pujol University Hospital, 08916 Badalona, Spain; garomerog.germanstrias@gencat.cat (G.R.-G.); nyrodriguezc.germanstrias@gencat.cat (N.R.-C.); ccanamerasf.germanstrias@gencat.cat (C.C.); jpaulma.germanstrias@gencat.cat (J.P.-M.); mitroya.germanstrias@gencat.cat (M.T.); jsoler.germanstrias@gencat.cat (J.S.-M.); fagraterol.germanstrias@gencat.cat (F.G.T.); mayasanchezb.germanstrias@gencat.cat (M.S.-B.); 2 REMAR-IGTP Group (Kidney-Affecting Diseases Research Group), Germans Trias i Pujol Research Institute (IGTP), 08916 Badalona, Spain; 3 International Renal Research Institute of Vicenza, 36100 Vicenza, Italy; 4 Nephrology Department, University Hospital Joan XXIII, 43005 Tarragona, Spain; m.urrutia.jou@gmail.com; 5 Nephrology Department, University Hospital Josep Trueta, IdIBGi Research Institute, Universitat de Girona, 17007 Girona, Spain; jcalabia.girona.ics@gencat.cat

**Keywords:** albuminuria, cardiorenal disease, chronic kidney disease, cardiovascular disease, heart failure

## Abstract

Kidneys have an amazing ability to adapt to adverse situations, both acute and chronic. In the presence of injury, the kidney is able to activate mechanisms such as autoregulation or glomerular hyperfiltration to maintain the glomerular filtration rate (GFR). While these adaptive mechanisms can occur in physiological situations such as pregnancy or high protein intake, they can also occur as an early manifestation of diseases such as diabetes mellitus or as an adaptive response to nephron loss. Although over-activation of these mechanisms can lead to intraglomerular hypertension and albuminuria, other associated mechanisms related to the activation of inflammasome pathways, including endothelial and tubular damage, and the hemodynamic effects of increased activity of the renin–angiotensin–aldosterone system, among others, are recognized pathways for the development of albuminuria. While the role of albuminuria in the progression of chronic kidney disease (CKD) is well known, there is increasing evidence of its negative association with cardiovascular events. For example, the presence of albuminuria is associated with an increased likelihood of developing heart failure (HF), even in patients with normal GFR, and the role of albuminuria in atherosclerosis has recently been described. Albuminuria is associated with adverse outcomes such as mortality and HF hospitalization. On the other hand, it is increasingly known that the systemic effects of congestion are mainly preceded by increased central venous pressure and transmitted retrogradely to organs such as the liver or kidney. With regard to the latter, a new entity called congestive nephropathy is emerging, in which increased renal venous pressure can lead to albuminuria. Fortunately, the presence of albuminuria is modifiable and new treatments are now available to reverse this common risk factor in the cardiorenal interaction.

## 1. Introduction

Albuminuria is prevalent in a spectrum of pathologies impacting both the kidneys and the heart. It has traditionally been associated as a marker of kidney disease, independent of GFR, particularly in patients with diabetes mellitus. Furthermore, in recent years it has transcended its role solely as a biomarker for injury, emerging as a therapeutic target [1]. Considering the introduction of novel medications that could potentially alter the natural course of cardiorenal diseases, it becomes imperative to comprehend the pathophysiology of albuminuria. This understanding is essential, not only for diagnostic purposes, but also for its application in the systematic monitoring and treatment of conditions that converge in cardiorenal diseases. 

Since the current description of the cardiorenal syndrome by Ronco et al. [2], the ongoing efforts to refine our understanding of the intricate interplay between the cardiovascular and renal systems, in both acute and chronic clinical scenarios, have given rise to an array of scholarly publications. Particularly noteworthy is the issuance of a position statement by the American Heart Association, which has significantly contributed to the discourse on this complex relationship in the medical domain [3]. 

The significant advancements in understanding the mechanisms implicated in the dysfunction of both organs have garnered substantial interest across multiple medical specialties, including nephrology, cardiology, and internal medicine. This growing interest seeks to expand the diagnostic and therapeutic strategies concerning the intricate interplay between the heart and the kidney. Consequently, there has been a noteworthy conceptual evolution in classical models utilized to elucidate acute or chronic damage to both organs. 

Illustrative examples of this evolution include the recognition that an elevation in creatinine during decongestive treatment in a patient with acute heart failure (AHF) may not invariably signify a structural change, but rather could be attributed to a functional disorder [4]. Similarly, there is a growing acknowledgment of the role of uremia and associated molecules in precipitating morphological and functional changes in the heart among patients with CKD [5,6]. 

Hence, certain authors posit the imperative identification of shared pathophysiological mechanisms underlying both functional and morphological alterations in both the heart and kidneys [7].

In conclusion, albuminuria is more than just a marker of cardiorenal risk or progression; its presence even in patients with normal GFR (blind spot theory) [8] obliges all specialties treating patients with cardiorenal syndrome to rapidly adopt this biomarker across the spectrum of diseases associated with acute and chronic cardiac dysfunction. 

The aim of this review is to describe the role of albuminuria in the cardiorenal interaction as a risk marker in acute and chronic heart failure and to describe new avenues of treatment. 

## 2. Conceptual Evolution of Albuminuria as a Cardiac and Renal Biomarker

### 2.1. Redefining Albuminuria

Albuminuria is defined as the abnormal loss of albumin in the urine. According to the nomenclature of kidney function and disease from the Kidney Disease Improving Global Outcomes (KDIGO) consensus conference [9], albuminuria is defined as a urinary albumin excretion rate (AER) >10 mg/d or an albumin–creatinine ratio (ACR) >10 mg/g. Previously, clinical guidelines published in 2012 for the evaluation and management of CKD preferred the use of albuminuria to proteinuria for diverse reasons, including the relationship between the presence of albuminuria and cardiovascular risk, the need to include albuminuria in the classification of CKD, and the fact that albumin is the major component of urinary protein in most kidney diseases [10]. The presence of albuminuria precedes the decline in glomerular filtration rate in most cases, and even in the presence of normal renal function, the presence of albuminuria is associated with a poor outcome. Considering the grading of albuminuria and its association with all-cause mortality, cardiovascular mortality, acute kidney injury (AKI), kidney failure (KF), and progressive CKD [10], three categories of albuminuria have been established: A1, which is normal or mildly elevated (ACR < 30 mg/g); A2, moderately elevated (ACR 30–300 mg/g); and A3. severely elevated (ACR >300 mg/g) [9] (Table 1). This classification is intended to facilitate the assessment of CKD severity and to determine individual cardiovascular risk according to GFR and the presence of albuminuria. A recent meta-analysis showed that the presence of albuminuria is associated with numerous cardiovascular and renal events, even in people with normal renal function as estimated by creatinine and cystatin C [11].

In addition, the nomenclature initiative suggests avoiding terms such as macroalbuminuria or microalbuminuria, which can lead to confusion about the size of the albumin in the urine. Finally, although there is a good correlation between simple untimed urine and 24-h urine albumin, the latter may not be accurate in clinical practice because of the difficulties some patients have in collecting urine [9]. 

### 2.2. Understanding the Pathophysiology of Albuminuria 

**Figure 1:**
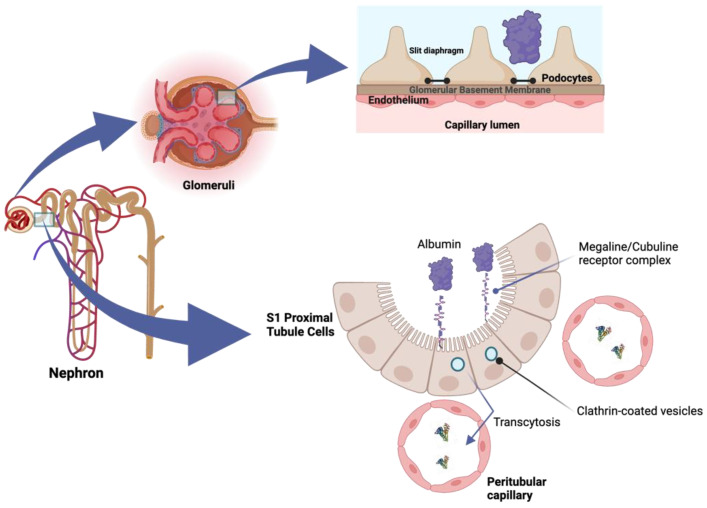
Albumin, filtered by the glomerular filtration barrier (GFB), is primarily reabsorbed in the early proximal tubule segments. This process involves clathrin-coated vesicles transcytosis mediated by the megalin–cubilin receptor complex. Once internalized, albumin undergoes lysosomal degradation, and the resulting products are recycled as free amino acids across the basolateral membrane, ultimately returning to the intravascular compartment.

Albumin is the main protein in the body, synthesized by hepatocytes and distributed mainly in the blood (10 to 15 g per day). Albumin serum half-life is 19 days; during the lifespan, the structure is modified by different reactions such as carbamylation and glycation [12,13,14,15]. The main functions of albumin are to maintain colloid pressure; it has antioxidant activity and is involved in the transport of endogenous substances (calcium, bilirubin, etc.) and exogenous substances such as drugs [13,14]. Albumin’s ability to transport drugs is particularly important in patients with AHF, as one of the mechanisms of diuretic resistance is related to hypoalbuminemia, which is associated with reduced diuretic delivery to the kidney [16]. In addition, albuminuria in these patients may bind to intratubular loop diuretics, reducing their activity. Renal albumin excretion is <6% of its synthesis (<0.2% of the normative daily albumin turnover of 10.5 g) and increased urinary albumin excretion is now not only a factor in the progression of CKD, but is also associated with the presence of cardiac pathology and is associated with poor outcomes in patients with HF [17]. Various pathways are involved in albumin catabolism, including endothelial cells, muscle, skin, liver, and proximal tubule cells in the kidney [18]. At the renal level, mechanisms such as glomerular filtration, proximal tubule reabsorption, and intracellular degradation play roles in the renal catabolism of albumin [18,19]. In normal circumstances, a minor quantity of albumin (approximately 100 mg per day) can be excreted in the urine [18]. Consequently, any impairment in glomerular filtration barrier (GFB) function or tubular injury may result in the presence of undesirable concentrations of albumin in the urine [19]. The ultrafiltration of water and small molecules is initiated in the GFB, comprising three layers: the inner layer of fenestrated endothelium, the glomerular basement membrane, and an outer layer of podocytes with their interdigitating processes. The disruption of any of these layers leads to a loss of selective glomerular ultrafiltration, permitting protein filtration [17,19]. While podocyte damage can arise from genetic mutations, it is frequently acquired [20,21]. An illustration of this is found in diabetic nephropathy, where hyperglycemic and hemodynamic factors can directly harm endothelial cells [17,19,20]. This process disrupts the glomerular basement membrane and causes damage to podocytes. Consequently, the physicochemical properties of the GFB are altered, leading to the loss of selectivity in the glomerular filtrate and allowing the passage of albumin [19,22]. It is now widely recognized that albumin, which undergoes ultrafiltration by the GFB, is primarily reabsorbed in the early segments of the proximal tubule [18,19,23]. The reabsorption of albumin occurs through clathrin-dependent endocytosis, mediated by the megalin–cubilin–amnionless receptor complex [18,23]. Once internalized into tubular cells, albumin undergoes lysosomal degradation and is subsequently recycled as free amino acids across the basolateral membrane, returning into the intravascular compartment [18,19,23] (Figure 1).

Alteration of the GFB leads to increased tubular reabsorption of albumin, which, in turn, is associated with increased mesangial activity and intrarenal complement activation, resulting in inflammation and tubular damage. This, in turn, triggers neurohormonal activation, increasing the activity of the renin–angiotensin–aldosterone system, which promotes sodium and water retention, resulting in volume overload [17,18,23]. The presence of albuminuria is often linked to the mechanism of hyperfiltration. This phenomenon can manifest as a supraphysiological increase in the GFR in kidneys with a normal number of functioning nephrons. Examples of such instances include healthy individuals experiencing a heightened glomerular filtration rate after a high protein intake or during pregnancy. Additionally, hyperfiltration may occur in patients with conditions such as obesity, diabetes mellitus, or autosomal dominant renal polycystic disease. It can also be observed in cases where there is a reduced number of nephrons [22,24]. In both scenarios—whether there is a normal or reduced number of nephrons—the objective is to elevate the GFR, resulting in glomerular hypertension. This increase in pressure may be correlated with albuminuria, glomerulosclerosis, and compromised renal function [17,24]. Other mechanisms that seem to be implicated in the development of albuminuria, especially in patients with AHF, involve increased central venous pressure (CVP) leading to systemic congestion, increased renal interstitial hydrostatic pressure, and dysfunction of the glomerular and systemic endothelium [25].

### 2.3. Albuminuria: The Neglected Biomarker

The traditional glomerulo-centric view has focused on diagnosing AKI and CKD primarily through the examination of creatinine levels and an estimated glomerular filtration rate (eGFR). Nonetheless, it is evident that creatinine, particularly in the context of AKI, serves as a late marker, becoming elevated only after previous tubular damage has occurred [26]. Additionally, reliance on eGFR alone may lead to the oversight of patients with chronic kidney disease, especially when the eGFR is greater than 60 mL/min/1.73 m^2^ [27].

The prevalence of albuminuria in the general population is estimated to range between 9.2% and 20.3% [17]. In individuals with HF, the incidence of proteinuria may be even higher, varying from 25% to 44% [17]. According to a recent cohort study involving 192,108 patients with hypertension or diabetes, the prevalence in this high-risk group was found to be 17.5% [28]. Interestingly, this study revealed that up to two-thirds of patients with albuminuria go undetected due to a lack of testing [28]. In fact, albuminuria is assessed in only 35% of patients with type 2 diabetes mellitus and 4% of patients with hypertension [27]. The low demand for the measurement of the ACR is truly alarming. It is well known that the presence of albuminuria, even at high levels within the normal range, is associated with an increased risk of all-cause mortality, progression of chronic kidney disease, and poor cardiovascular health [17,27,29]. In fact, there are patients who exhibit some degree of albuminuria and have an estimated glomerular filtration rate >60 mL/min/m2, and they are more likely to experience cardiovascular impairment. This phenomenon has recently been termed the ‘blind spot’ [8,27]. The significance of albuminuria as a risk marker is acknowledged as one of the five pillars in the European Society of Cardiology’s guidelines for preventing cardiovascular disease [30,31].

## 3. Albuminuria in the Continuum of Cardiorenal Disease

**Figure 2:**
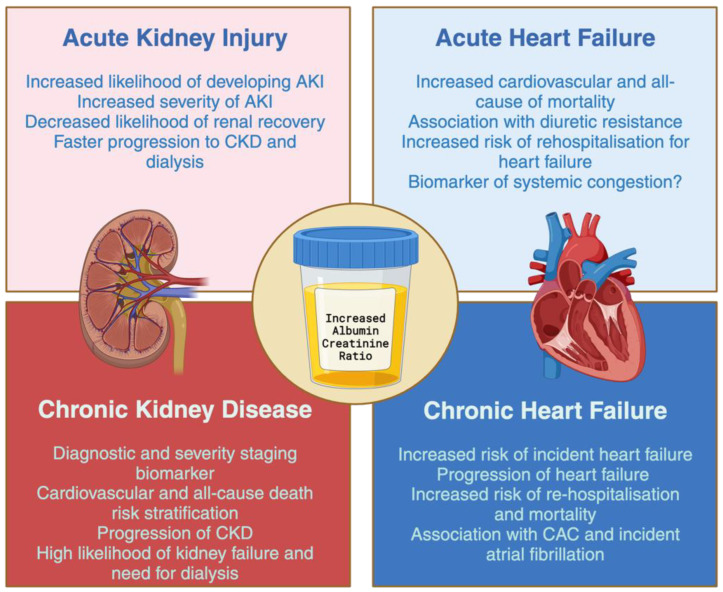
Albuminuria-associated cardiovascular and renal outcomes across the spectrum of renal and cardiac disease. Abbreviations: AKI—acute kidney injury; CKD—chronic kidney disease; CAC—coronary artery calcification.

The incorporation of albuminuria into the diagnostic of CKD has facilitated the creation of a risk chart outlining the progression of CKD [10] and the risk of premature all-cause and cardiovascular mortality [10,32]. Nonetheless, the existence of albuminuria may be linked to diverse negative consequences, including the onset of HF, and these events may manifest throughout the spectrum of both kidney and heart disorders [17,27,33] (Figure 2).

### 3.1. Albuminuria and Acute Kidney Injury

The progression in understanding the diagnosis of AKI acknowledges it as a syndrome wherein one encounters the coexistence of one or more mechanisms causing kidney damage [34]. Nevertheless, there exist diverse risk factors that can potentially contribute to the onset or advancement of AKI [35]. An instance of this is CKD, where a diminished GFR significantly elevates the likelihood of experiencing AKI [36]. Notably, recent findings reveal that albuminuria stands out as another contributor that amplifies the risk of AKI, and this connection demonstrates a linear pattern, persisting even at levels surpassing the upper limit of normal ACR (10–29 mg/g) [37]. The most compelling support for this connection is derived from an extensive study involving 920,985 individuals in Alberta, Canada. Out of this cohort, 6520 participants experienced AKI. The research revealed that both mild proteinuria (trace or urine dipstick reading of 1+) and severe proteinuria (urine dipstick ≥ 2+) were associated with a heightened risk of AKI across all eGFR values, with the exception of individuals with end-stage CKD and those undergoing dialysis [38]. In a recent meta-analysis conducted by James et al., findings were consolidated from eight cohorts. The results indicated a heightened likelihood of AKI linked to the existence of albuminuria, irrespective of the presence of diabetes [39]. This association has been observed notably following different interventions, including the administration of contrast agents [40], cardiac and non-cardiac surgical procedures [41,42,43,44,45], and in clinical situations such as sepsis [46] or acute myocardial infarction [47]. Moreover, the existence of albuminuria is linked to more pronounced AKI and a diminished probability for kidney recovery [48,49].

Once AKI is diagnosed, the existence of albuminuria has been linked to the deterioration of AKI stages [50,51]. Moreover, instances of AKI have been correlated with the initiation or exacerbation of proteinuria, placing these individuals at elevated risk for the advancement of CKD. In summary, proteinuria has been associated with increased risk of AKI, more severe and progression of AKI, less recovery of kidney function and greater progression of CKD and dialysis dependence.

### 3.2. Albuminuria and Chronic Kidney Disease

Albuminuria plays a crucial role in both diagnosing and stratifying the risk for patients dealing with CKD [10]. Due to its nephrotoxic and proinflammatory properties, which can lead to apoptosis and fibrosis, albuminuria is a risk factor for further progression of kidney damage, progression of CKD, and the associated cardiovascular risk [52,53,54]. Employing tools like eGFR and ACR enables the targeted allocation of resources, aiding in the early initiation of preventive measures or the referral of high-risk CKD patients [55]. Individuals with an ACR exceeding 300 mg/g exhibit a heightened annual loss of glomerular filtration rate compared to the diabetic reference group [56]. Various risk assessment tools, including the kidney failure risk equation, have emerged [57]. This equation incorporates factors such as ACR, eGFR, age, and sex to forecast the likelihood of KF at both the 2- and 5-year marks [57]. Originating from a Canadian cohort of patients with CKD stages G3–G5 referred to nephrologists, this calculator has undergone validation in over 700,000 individuals across 30-plus countries [58,59]. Notably, the inclusion of albuminuria in the calculator’s development significantly enhanced its predictive accuracy for kidney disease progression when compared to conventional variables such as age, sex, hypertension, diabetes, blood pressure, and body weight [57].

Ultimately, it is crucial to acknowledge that albuminuria serves as an indicator of kidney damage and a forecaster of the progression of CKD. Therefore, diminishing albuminuria should be employed as a substitute measure for CKD advancement in clinical trials aiming for novel primary endpoints that are more straightforward to comprehend than the conventional major adverse renal events. Clearly, the mitigation of albuminuria has emerged as a fundamental aspect of treating CKD [60,61].

### 3.3. Albuminuria and Congestive Acute Heart Failure

Historically, there has been acknowledgment of the presence of mild proteinuria in individuals experiencing AHF [62], and, in numerous instances, the proteinuria demonstrated improvement following the initiation of decongestive therapy [63]. Nevertheless, the precise significance of albuminuria in patients with AHF remains to be definitively determined. In individuals experiencing AHF, renal dysfunction might arise as a consequence of elevated central venous and right atrial pressures [64]. Contrary to prior assumptions suggesting anterograde transmission, these pressures are now understood to be transmitted in a retrograde way [65]. The heightened venous pressure results in a reduction of renal perfusion pressure, prompting the activation of autoregulation mechanisms [66]. These mechanisms strive to augment the filtration fraction in an effort to uphold a constant GFR, leading to a state of hyperfiltration [67]. Subsequently, this hyperfiltration induces an upswing in the tubular reabsorption of water and sodium. The consequence is an escalation in the hydrostatic pressure within the renal interstitium. Unfortunately, the renal organ lacks the capacity to adequately respond to volume overload, primarily due to the presence of the kidney capsule. This condition has recently been recognized as kidney tamponade [68]. The cumulative effect manifests as tubular injury, facilitated by the activation of neurohormonal mechanisms, inflammation, and oxidative stress. This intricate process is now referred to as congestive nephropathy [69].

An established association exists between the presence of albuminuria and increased hospitalizations for HF. Boorsma et al. demonstrated a high prevalence of macroalbuminuria (10%) and microalbuminuria (35%) in HF patients [70]. In both the index and validation cohorts, worsened New York Heart Association class, higher loop diuretic dose requirements, increased clinical and echocardiographic signs of congestion, greater diastolic dysfunction, and elevated levels of congestive biomarkers such as NT-proBNP, bio-adrenomedullin, and carbohydrate antigen 125 were observed [70]. Linear regression analyses also revealed positive correlations with biomarkers of tubular injury such as KIM-1 and NGAL [70]. Mechanisms related to congestive kidney disease, including retrogradely transmitted increased central venous pressure, may account for the presence of albuminuria. Additionally, elevated renal interstitial hydrostatic pressure and endothelial dysfunction contribute to alterations in the glycocalyx, resulting in significant saturation of the glycosaminoglycan network. This alteration in function allows small increases in capillary hydrostatic pressure to lead to the formation of edema [25].

### 3.4. Albuminuria and Heart Failure

The intricate connections between albuminuria and the development or progression of HF involve diverse and intricate mechanisms [17]. These mechanisms primarily revolve around endothelial damage, tubular injury, and the existence of concurrent conditions (e.g., diabetes mellitus, hypertension, obesity) linked to inflammation. These conditions contribute to tissue and vascular congestion, triggering the activation of neurohormonal processes and the renin–angiotensin–aldosterone system [17,27]. Moreover, endothelial dysfunction is implicated in hastening atherosclerosis by fostering oxidative stress [71]. The manifestation of albuminuria is linked to a 1.7–2.7-fold heightened risk of HF development [72,73], even in cases where the eGFR appears normal [74]. Furthermore, albuminuria’s presence correlates with HF progression, irrespective of phenotype, and is associated with elevated rates of hospitalization and mortality [75,76]. These outcomes persist regardless of the coexistence of comorbidities such as type 2 diabetes mellitus or hypertension. The HOPE study revealed a direct correlation between an elevated ACR and the risk of hospitalization [77]. Each 0.4 mg/mmol increase in the ratio escalates the risk of hospitalization by 11%, a risk that remains evident even at low levels of albuminuria (>8 mg/g) [29,77].

The impact of albuminuria on atherosclerosis has been previously documented. Research conducted in Korea, involving 45,006 participants with no history of coronary artery calcification, revealed a connection between the existence of albuminuria and the presence of coronary artery calcification as determined through coronary computed tomography [78]. Additionally, findings from the TRACER trial [79] indicated that the presence of albuminuria correlated with a noteworthy rise in mortality among individuals experiencing acute coronary syndromes (HR: 1.65; 95% CI: 1.15–2.37). Importantly, this increased mortality remained independent of the eGFR.

Albuminuria is not only associated with morphological or functional changes in the heart; a recent meta-analysis has shown that individuals with albuminuria face an elevated risk of developing incident atrial fibrillation. Moreover, the risk is further heightened when ACR levels exceed 30 mg/g [80].

Finally, the use of albuminuria as a biomarker of kidney damage allows early identification of individuals at risk of developing or progressing HF, before the expected decline in GFR associated with adverse outcomes. This provides various medical specialties, particularly cardiology, with an additional valuable urinary biomarker for both diagnosis and treatment monitoring. It also provides potential links for future clinical trials in HF treatment.

## 4. Minimizing Albuminuria: Old and New Approaches

The renin–angiotensin system (RAS) inhibitors currently serve as the cornerstone of treatment for slowing the progression of CKD [81,82,83]. Their use is advocated in various treatment guidelines for conditions such as CKD [10], diabetes mellitus [84], hypertension [85], and HF [86]. Unfortunately, a significant percentage (40–50%) of patients may not respond adequately to treatment with RAS inhibitors [87]. Interestingly, new treatment approaches have recently emerged, demonstrating significant efficacy in reducing albuminuria and slowing CKD progression, even in settings where there were previously few therapeutic options, such as IgA nephropathy [88]. These pharmacological groups include sodium–glucose cotransporter-2 inhibitors (SGLT2i), glucagon-like peptide 1 receptor agonists (GLP1-RA), mineralocorticoid receptor antagonists (MRA), endothelin receptor antagonists, and Janus kinase (JAK-STAT) inhibitors. Despite the use of traditional drugs such as RAS inhibitors and SGLT2i, the risk of progression in CKD patients remains unacceptably high. Therefore, early detection through the measurement of the albumin-to-creatinine ratio (ACR), early introduction of older drugs, and appropriate rotation through different drug classes [89] will play a pivotal role in avoiding kidney failure, the need for dialysis, or worse, excess cardiovascular morbidity and mortality.

### 4.1. RAS Inhibitors

The albuminuria-reducing effect of this pharmacological group has been distinctly demonstrated in numerous studies [81,82,83,90,91,92]. One of the initial published articles utilizing enalapril exhibited a notable decrease in albuminuria measured in 24-h urine compared to the placebo group, which experienced a significant increase [93]. This increase was associated with the stabilization of creatinine levels. Another study involving irbesartan indicated a reduction of more than 30% in urinary albumin excretion within the intervention group [83]. Importantly, this benefit was independent of blood pressure control. It is noteworthy that both angiotensin-converting enzyme (ACE) inhibitors and angiotensin II receptor blockers (ARBs) demonstrate similar efficacy. The DETAIL study, which compared enalapril with telmisartan, found no significant changes in estimated glomerular filtration rate (eGFR) or urinary albumin excretion [94]. However, the nephroprotective effect of the renin–angiotensin system (RAS) seems to hinge on the presence of proteinuria [95], as not all patients experience a significant reduction in ACR.

### 4.2. Sodium–Glucose Cotrasporter-2 Inhibitors

SGLT2 inhibitors (SGLT2i) slow the progression of chronic kidney disease [96]. Originally developed as oral hypoglycemic agents, these drugs demonstrate a significant cardiorenal protective effect with minimal impact on glycemic control [96,97,98]. Interestingly, the cardioprotective and renoprotective effects persist even in non-diabetic patients. Furthermore, these medications have proven effective in preventing heart failure development and reducing cardiovascular risk in individuals both with and without diabetes mellitus. The mechanism of action for this drug class involves inhibiting SGLT2 in the proximal tubule, enhancing glucose and sodium excretion [99]. However, the precise underlying mechanism responsible for the cardiac and renal benefits remains not fully understood. A post hoc analysis of the EMPA-REG trial [100] shows a noteworthy 18% reduction ACR at week 12, compared to a placebo, and was associated with a decreased long-term risk of cardiovascular and renal outcomes. The DAPA-CKD trial [101], which examined both the reduction in albuminuria severity and renal cardiovascular (RCA) progression, revealed a significant 35.1% reduction in ACR for patients with type 2 diabetes mellitus and 14.8% for those without type 2 diabetes mellitus. A post hoc analysis of the CREDENCE trial [102] investigated the impact of canagliflozin on albuminuria, demonstrating a significant 31% reduction at week 26 of canagliflozin treatment, independently associated with decreased long-term renal and cardiovascular outcomes. A recently published meta-analysis indicates that the renal benefits of SGLT2i appear to be independent of the baseline estimated glomerular filtration rate (eGFR). Interestingly, the reduction in renal events was more pronounced in patients with any degree of albuminuria [103]. Unlike renin–angiotensin system (RAS) inhibitors, the renal protection provided by SGLT2i is not contingent on baseline albuminuria [103].

### 4.3. Glucagon-Like Peptide 1 Receptor Agonist

GLP-1 receptor agonists (GLP-1RAs) are anti-diabetic agents with the potential to decelerate the progression of chronic kidney disease (CKD) by inhibiting oxidative stress, fibrosis, and apoptosis [104]. These medications reduce gastric emptying, lower glucagon levels, and regulate appetite by decreasing blood glucose levels and body weight [105]. Renally, GLP-1RAs seem to facilitate natriuresis and mitigate hyperfiltration [104]. According to a recent meta-analysis encompassing at least 22 published articles [106], the use of GLP-1RAs was linked to a noteworthy reduction (16.14%) in albuminuria in patients with type 2 diabetes mellitus compared to a placebo. A novel compound, tirzepatide, which acts as both a glucose-dependent insulinotropic polypeptide (GIP) receptor agonist and a GLP-1 RAs, exhibited a significant impact on decreasing albuminuria [107]. A recently published meta-analysis [108] indicates that the utilization of tirzepatide was correlated with a substantial reduction in ACR compared to controls (−26.9%). This effect was most prominent at ACR levels >30 mg/g, although no significant impact on creatinine clearance was observed in the same meta-analysis.

### 4.4. Mineralocorticoid Receptor Antagonist

Spironolactone and eplerenone are two steroidal mineralocorticoid receptor antagonists (MRAs) traditionally used as coadjuvants to reduce proteinuria [109]. Spironolactone has demonstrated a significant proteinuria reduction (61%), and this effect is enhanced when combined with RAS inhibitors [110]. Similarly, the reduction of albuminuria is observed with the use of eplerenone [111]. However, these drugs are relatively or strongly contraindicated in patients with advanced CKD, in part due to the increased risk of hyperkalemia [112,113]. Recently, a new non-steroidal MRA, finerenone, has emerged. It exhibits greater selectivity for the mineralocorticoid receptor compared to spironolactone and eplerenone [109,114]. The ARTS clinical trial revealed that finerenone reduced baseline ACR by at least 50%, with a lower incidence of hyperkalemia and less deterioration in renal function [115,116]. A post hoc analysis of the Fidelio [117] and Figaro [118] trials showed a mean reduction in ACR at month 4 of 33.6% in patients treated with finerenone, compared to only 2.6% in the placebo group [119]. Additionally, a significantly greater reduction in cardiovascular and renal events was observed in patients with a greater than 30% reduction in ACR [119]. Furthermore, there was an additive effect with the combination of finerenone and dapagliflozin, demonstrating a greater reduction in albuminuria with the combination therapy [120]. Indeed, finerenone is currently recommended for use in patients with type 2 diabetes mellitus and persistent albuminuria [121].

## 5. Conclusions

By strengthening our diagnostic approach, albuminuria is emerging not only as a cornerstone in the identification of CKD, but also as a beacon illuminating the way to a broader understanding of individual risk, particularly in the areas of cardiovascular and all-cause mortality. Despite its myriad benefits, this invaluable biomarker remains underappreciated, overshadowed by the misconception that a normal eGFR equates to the absence of kidney disease. Albuminuria’s reach extends far beyond nephrology, becoming a key player in unravelling the intricate dance of cardiorenal interactions. In every clinical scenario, it becomes a signpost revealing the looming specter of HF or AKI, its presence serving as an ominous marker of increased mortality. In addition, the simplicity, practicality, and reliability of spot UACR (compared to 24-h urine collection) make it an easy test to implement in both inpatient and outpatient settings in any medical specialty. The real question that beckons is the following: Why not harness its power for primary prevention, even in the absence of advanced heart or kidney disease? Let us embrace the potential of albuminuria as a preventive biomarker, crossing boundaries and lighting the way to proactive well-being. 

## Figures and Tables

**Table 1:** Current definition of albuminuria and proteinuria using spot albuminuria-to-creatinine ratio (ACR), spot proteinuria-to-creatinine ratio (PCR), and urine protein dipstick. Adapted from [9].

Degree of Albuminuria/Proteinuria	Normal to Mildly Increased	Moderately Increased	Severely Increased
Spot ACR	<30 mg/g	30–299 mg/g	≥300 mg/g
Spot PCR	<150 mg/g	150–499 mg/g	≥500 mg/g
Urine protein dipstick	Negative to trace	Trace to +	+ or greater

## Data Availability

Not applicable.
